# An Energy-Economy-Environment Model for Simulating the Impacts of Socioeconomic Development on Energy and Environment

**DOI:** 10.1155/2014/353602

**Published:** 2014-02-11

**Authors:** Wenyi Wang, Weihua Zeng, Bo Yao

**Affiliations:** School of Environment, Beijing Normal University, No. 19, XinJieKouWai Street, HaiDian, Beijing 100875, China

## Abstract

Many rapidly developing regions have begun to draw the attention of the world. Meanwhile, the energy and environmental issues associated with rapid economic growth have aroused widespread critical concern. Therefore, studying energy, economic, and environmental systems is of great importance. This study establishes a system dynamic model that covers multiple aspects of those systems, such as energy, economy, population, water pollution, air pollution, solid waste, and technology. The model designed here attempts to determine the impacts of socioeconomic development on the energy and environment of Tongzhou District in three scenarios: under current, planning, and sustainable conditions. The results reveal that energy shortages and water pollutions are very serious and are the key issues constraining future social and economic development. Solid waste emissions increase with population growth. The prediction results provide valuable insights into social advancement.

## 1. Introduction

Tongzhou District is one of three new towns in Beijing [1]. In recent years, the economy has grown quickly, and the process of urbanization has accelerated rapidly. However, energy shortages and environmental pollution have been aggravated as the economy has grown, conflicting seriously with our efforts at environmental protection. There are certain problems in the energy and water sectors of Tongzhou District that have aroused concerns from the greater society.

System dynamics is a theory of system structure, an approach to understanding complex systems and a method for analyzing system dynamic behavior that explores, assesses, and prognosticates the impacts in a holistic manner [2]. It promotes a more complicated, quantitative simulation and is able to achieve more robust and reliable outcomes [3]. Since the 1960s, several studies have used the system dynamic method to address or simulate scenarios in many different applications, such as in socioeconomic dynamics [4, 5], business systems [6], corporate planning and policy design [7], urban dynamics [8, 9], analysis of urban problems and responses to policy changes [10], agricultural systems [11], agricultural policy analysis [12], ecological systems [13], predicting flood patterns caused by snowmelt [14], water resources management [15], simulation of recharge and flow mechanisms in a fractured bedrock aquifer [16], simulating the process of accumulated metals treatment in constructed wetlands [17], implementing health care policies and programs [18], analyzing the impact of strategies for addressing epidemics, [19], environmental systems [20, 21], prediction of solid waste generation [22], analysis of collection capacity and electricity generation from solid waste [23], and even as a decision support tool to solve the coastal zone management problem [24].

In the field of energy-economy-environment systems research, contributions using system dynamics have seen applications in environmental impact analysis [25], global environmental development [26], ecoindustrial systems planning [27, 28], dynamic assessment of urban economy-resource-environment systems [29], and economic and environmentally sustainable development [30]. This paper is supposed to predict the impacts of socioeconomic development on the energy and environment of Tongzhou District under a business-as-usual scenario, a planning-oriented scenario, and a sustainable development scenario by proposing an improved SD model in Stella; this paper further aims to identify the key factors constraining the future advancement of Tongzhou District. The improved SD model is based on Guo's model [31, 32], which enhances necessary factors to allow for more comprehensive analysis.

## 2. Study Area and Data Sources

### 2.1. Site Description

**Figure 1:**
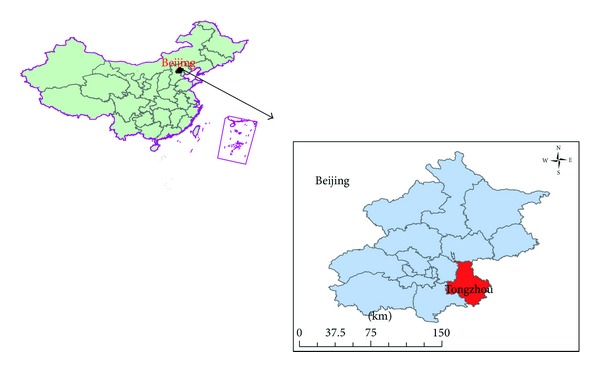
Location of Tongzhou District, Beijing City, China.

Tongzhou District is located in the southeast part of Beijing (Figure 1), at the east gate of the capital, is the node linking the multiple development corridors of the outskirts of Bohai, is the starting point of Jing-Hang Grand Canal, and is 20 km from Tiananmen Square. Tongzhou is roughly 37 km wide from east to west and 48 km from north to south. It has an area of approximately 906 square kilometers, which makes up roughly 5% of Beijing's total territory and 14% or so of Beijing's plains [33].

### 2.2. Data Sources

The data include socioeconomic, pollutant discharge, environmental protection, and energy consumption data and were collected from the Tongzhou statistical yearbook, the Tongzhou new town plan, industry development planning (2006–2010), population development and control planning (2006–2010), the Beijing city general plan (2004–2020), the pollution discharge declaration of Tongzhou District (2001–2010), environmental statistics from Tongzhou District (2001–2010) and environmental protection planning.

## 3. Methods

### 3.1. Description of the Model

**Figure 2:**
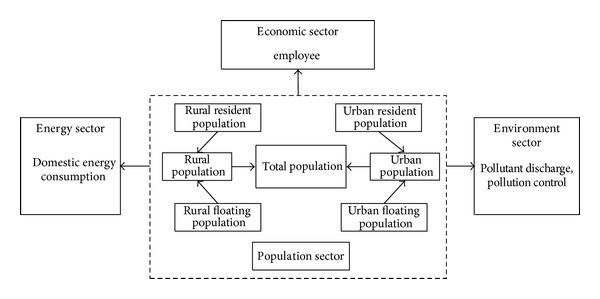
The population sector and the relationship with other sectors.

**Figure 3:**
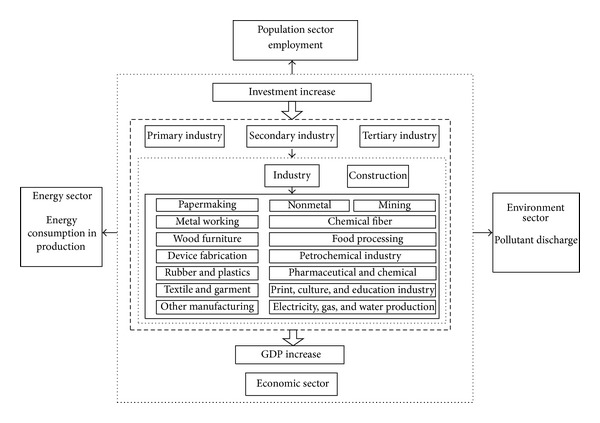
The economy sector and the relationship with other sectors.

**Figure 4:**
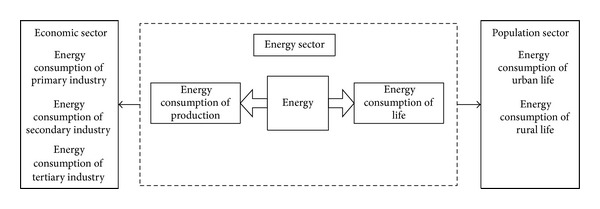
The energy sector and the relationship with other sectors.

**Figure 5:**
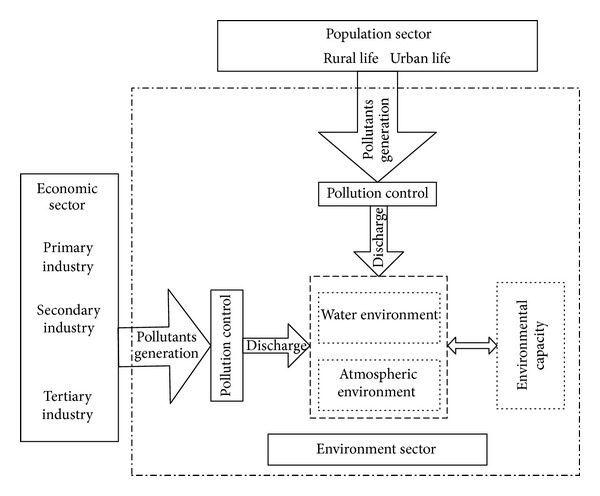
The environment sector and the relationship with other sectors.

The model was divided into four parts: population, economy, energy, and environment. The structure of the population sector and its relationship with other sectors are shown in Figure 2. In the model, the total population was divided into two parts: urban and rural populations, which both consisted of the resident and floating populations. The floating population included short-term workers, tourists, and students. The structure of the economic sector and its relationship with other sectors is shown in Figure 3. In the model, the economic sector was divided into three modules, primary, secondary, and tertiary industry, in accordance with the socioeconomic statistical classification. Among these, secondary industry included industry and construction. Industry could also be divided into 15 sub-industries in the design of the model structure. These sub-industries were: mining (I01), food processing (I02), textiles and garments (I03), wood furniture (I04), papermaking (I05), printing, culture, and education (I06), petrochemicals (I07), pharmaceutical and chemical (I08), chemical fibers (I09), rubber and plastics (I10), nonmetal processing (I11), metal processing (I12), equipment manufacturing (I13), other manufacturing (I14) and electricity, and gas and water production (I15). The energy sector's structure and its relationship with other sectors are shown in Figure 4. Energy demand covered energy consumption in production and in daily life. The environment sector's structure and its relationship with other sectors are shown in Figure 5. In the process of human life and production activities, sewage, waste gas, and solid waste are constantly discharged to the environment.

**Figure 6:**
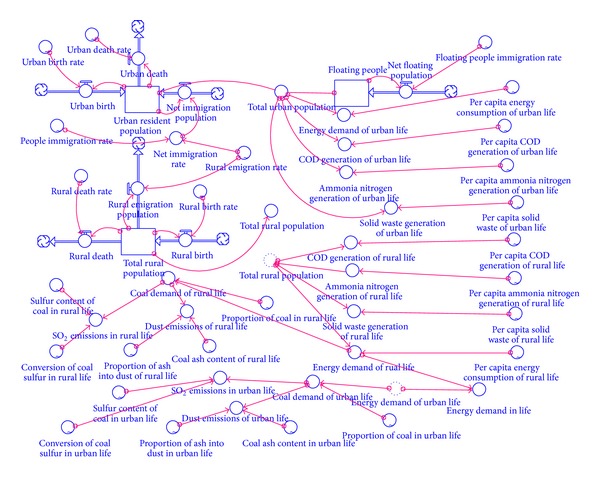
The main part of the system dynamic model (population module).

**Figure 7:**
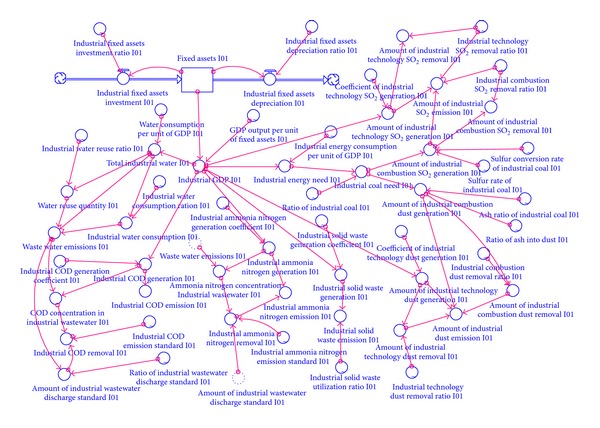
The main part of the system dynamic model (industry I01 module).

The model was written in Stella with a time step of 1 year and a model run spanning 10 years. The boundary of the Tongzhou SD model was the Tongzhou District in Beijing. The model illustrates the connections between the economy, the population, energy, and the environment and can be used to show the isolated effects of individual variables. As the main parts of model, the stock-flow diagrams of the population module and the industry module (taking I01 as an example) are shown in Figures 6 and 7.

### 3.2. Model Evaluation 

#### 3.2.1. Model Calibration

The model was calibrated by testing the parameters of historical fit using the correction coefficient (*R*), absolute relative error (ARE), and mean absolute relative error (MARE) ((1)–(3))[34, 35]:

(1) $$\begin{array}{c} R=\frac{\sum_{t=1}^{n}\left(Y_{t}-\bar{Y_{t}}\right)\left(\hat{Y_{t}}-\bar{\hat{Y_{t}}}\right)}{\sqrt{\sum_{t=1}^{n}{\left(Y_{t}-\bar{Y_{t}}\right)}^{2}\sum_{t=1}^{n}{\left(\hat{Y_{t}}-\bar{\hat{Y_{t}}}\right)}^{2}}}, \end{array}$$

where *t* is the time unit, *n* is number of units of data, *Y*_*t*_ and $\hat{Y_{t}}$ represent observed and simulated results, and $\bar{Y_{t}}$ and $\bar{\hat{Y_{t}}}$ represent the means of the observed and simulated results.

#### 3.2.2. Model Validation

Model validation is the comparison of model results with independent observations. ARE and MARE are also used for model performance validation [34, 36].

(2) $$\begin{array}{c} \text{ARE}=\left|\frac{\left(\hat{Y_{t}}-Y_{t}\right)}{Y_{t}}\right|, \end{array}$$

(3) $$\begin{array}{c} \text{MARE}=\frac{1}{n}\overset{n}{\underset{t=1}{\sum }}\left|\frac{\left(\hat{Y_{t}}-Y_{t}\right)}{Y_{t}}\right|. \end{array}$$

#### 3.2.3. Sensitivity Analysis

The sensitivity analysis was conducted using the univariate method, which varies the value of one parameter at a time while keeping the values of other parameters constant. The sensitivity index can be calculated using the following (4) [34, 37]:

(4) $$\begin{array}{c} S_{Y}=\left|\frac{dY_{t}}{Y_{t}}\cdot \frac{X_{t}}{dX_{t}}\right|, \end{array}$$

where *t* is time, *S*_*Y*_ represents the sensitivity index of system state *Y* to parameter *X*, *Y*_*t*_ denotes the system state at time *t*, *X*_*t*_ is the value of the system parameter at time *t*, and *dY*_*t*_ and *dX*_*t*_, respectively, are the values for a change in system state *Y* and parameter *X* at time *t*.

The general sensitivity degree index is the degree of sensitivity of a parameter to the *n* stock variables (*Y*_1_, *Y*_2_,…, *Y*_*n*_) at time *t*, which is defined by [34]

(5) $$\begin{array}{c} S=\frac{1}{n}\overset{n}{\underset{i=1}{\sum }}S_{Yi}, \end{array}$$

where *S* is the general sensitivity degree, *n* denotes the number of stock variables, and *S*_*Yi*_ is the sensitivity degree of stock variable *Y*_*i*_.

### 3.3. Scenario Establishment 

*(1) Business-as-Usual Scenario*. In the business-as-usual scenario, energy, economic, and environmental developments remain in accordance with the current trends without any constraints. The purpose of the business-as-usual scenario is to analyze the gap between the scale of energy-economic-environmental development and the goals of the Tongzhou new town plan.

*(2) Planning-Oriented Scenario*. The planning-oriented scenario refers to the possible states of energy, the economy, and the environment under the constraints of regional planning objectives. The planning-oriented scenario is designed to determine the energy consumption and the pollution emissions entailed by realizing the social and economic goals of Tongzhou's region planning.

*(3) Sustainable Development Scenario*. The sustainable development scenario is an idealized scenario that can maximize the regional economic development objectives and reduce the enormous pressure on energy and the environment through advancing a circular economy and increasing investments in environmental protection. The designs of the population size and the scale of the economy remain consistent with those in the planning-oriented scenario.

## 4. Results and Discussion

### 4.1. Model Evaluation Results

#### 4.1.1. Calibration and Validation Results

The data series during the period of 2004 to 2010 were divided into two parts to conduct model calibration and validation; the first part, from 2004 to 2008, was used for model calibration, and the rest were used for validation.

**Figure 8:**
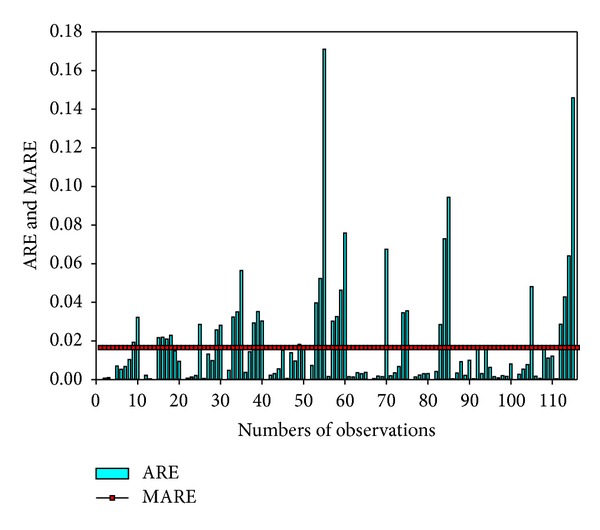
Calibration results using absolute relative error (ARE) and mean ARE (MARE).

**Figure 9:**
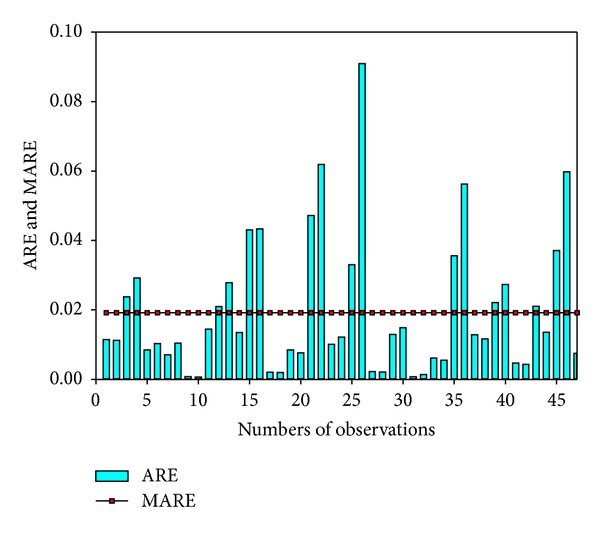
Validation results using ARE and MARE.

The correlation coefficient (*R* = 0.91) between the observed and simulated results was high following the calculation of (1), and the MARE of 0.017 was low (Figure 8), which indicated that the model results corresponded well with historical observations. The validation results are illustrated in Figure 9, in which the low ARE and MARE (0.019) indicate that the model had very good performance in reflecting the behavior of the system.

#### 4.1.2. Sensitivity Analysis

In the system dynamic model, 15 parameters had high levels of uncertainty, including industrial COD generation coefficients (I01, I05, and I09), industrial solid waste generation coefficients (I01, I08, and I15), industrial ammonia nitrogen generation coefficients (I05, I07, and I08), industrial energy consumption per unit of GDP (I05, I10, and I11) and sulfur conversion rates for industrial coal (I10, I14, and I15).

The sensitivity analysis results are illustrated in Table 1. It can be seen that the sensitivity degrees of the industrial COD generation coefficients (I05, I08) were very high. The system state was also sensitive to the parameters of industrial solid waste generation coefficients (I01, I08) and industrial ammonia nitrogen generation coefficients (I05). The sensitivity values imply that the simulation results can be significantly affected by the errors in the values of these parameters. The parameters of industrial energy consumption per unit of GDP (I05, I10, and I11) were less sensitive, and none of the remaining parameters were sensitive to target system state.

### 4.2. Simulation Results

#### 4.2.1. Simulation Results of the Business-as-Usual Scenario

**Figure 10:**
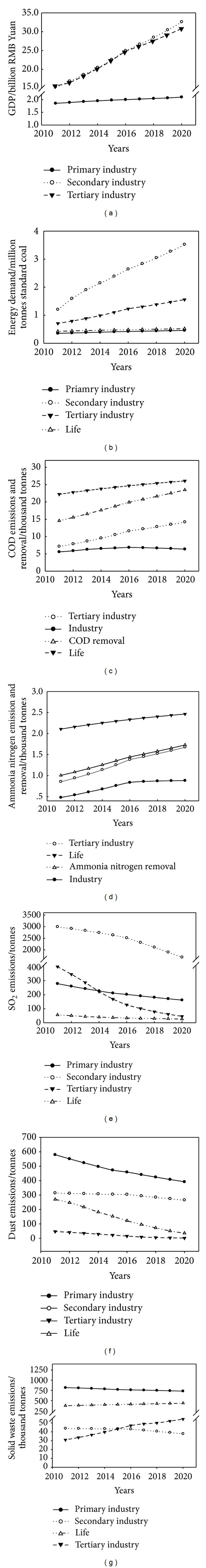
Simulation results under the business-as-usual scenario. (a) Economy development, (b) energy consumption, (c) COD emissions, (d) NH_3_-N emissions, (e) SO_2_ emissions, (f) dust emissions, and (g) solid waste emissions.

*(1) Economic Development.* In the business-as-usual scenario, the comprehensive economic strength of Tongzhou District continues to expand,primary industry grows steadily, secondary industry increases rapidly to take a leading role, and tertiary industry grows on an annual basis. According to the forecast results shown in Figure 10(a), Tongzhou District's GDP in 2020 reaches 65.55 billion RMB Yuan, an increase of 99.8 percent compared with that in 2011. The output values of the three degrees of industry were 2.10, 32.62, and 30.83 billion RMB Yuan, and their proportions were 3, 50, and 47 percent, respectively. According to the new town plan, the GDP should reach at least 120 billion RMB Yuan, and thus economic growth under the business-as-usual scenario cannot meet the planning target requirements.

*(2) Energy Consumption.* From the forecast results shown in Figure 10(b), there is a large increase in the energy demand of Tongzhou in the next 10 years. The total energy demand in 2020 is 6.07 million tonnes standard coal. Secondary and tertiary industries are the main energy-consuming sectors, with energy consumption of, respectively, 3.53 and 1.56 million tonnes standard coal in 2020. Additionally, secondary and tertiary industry energy consumption proportions increase continuously, accounting for, respectively, 58 and 26 percent in 2020. Primary industry and daily life require relatively small quantities of energy, with consumption of, respectively, 0.46 and 0.52 million tonnes standard coal in 2020.

Tongzhou District cannot be self-sufficient in energy; the energy supply mainly relies on energy distributions from China and Beijing. However, according to the new town planning requirements, energy consumption per unit of GDP in 2020 should reach 0.43 tce/10000 RMB. In the business-as-usual scenario, that measure is 0.93 tce/10000 RMB. Thus, the energy supply of Tongzhou District cannot satisfy the needs of future daily life and production.

*(3) Water Pollution Emissions.* In the business-as-usual scenario, COD and ammonia nitrogen emissions from all of the industries in Tongzhou District rise over the next decade, especially from tertiary industry. According to the simulation results illustrated in Figures 10(c) and 10(d), the production amounts of COD and ammonia nitrogen in wastewater increase to, respectively, 46.65 and 5.03 thousand tonnes in 2020. Subsequently, the wastewater reaches the sewage treatment plant and is treated before it can be released. The COD (ammonia nitrogen) removal numbers in Figures 10(c) and 10(d) indicate the COD (ammonia nitrogen) removal amounts in the treatment process. Specifically, the actual emissions of COD and ammonia nitrogen will be, respectively, 23.19 and 3.30 thousand tonnes in 2020. Domestic sewage is still a major source of water pollution, with production amounts of COD and ammonia nitrogen at 26.08 and 2.47 thousand tonnes, respectively. Furthermore, the proportions of COD and ammonia nitrogen discharge from tertiary industry increase by 10 and 9 percent in 10 years, thus making tertiary industry a main source. Industrial COD and ammonia nitrogen production increases somewhat to 6.36 and 0.88 thousand tonnes, respectively, in 2020.

The environmental water capacity of COD is 3.56 thousand t/a, and for ammonia nitrogen the amount is 0.38 thousand t/a [38]. With the increase in water pollution in Tongzhou District, water quality will necessarily deteriorate.

*(4) Air Pollution Emissions.* From the results shown in Figures 10(e) and 10(f), SO_2_ and dust emissions are 1.92 and 0.70 thousand tonnes, respectively, in 2020. The SO_2_ emissions from primary and secondary industry make up 8 and 88 percent of the regional total in 2020, respectively, 0.16 and 1.68 thousand tonnes. However, dust emissions from primary and secondary industry are, respectively, 0.39 and 0.27 thousand tonnes in 2020, accounting for 56 and 38 percent of the regional total. The air pollution emissions continue to decline in the long term, and the SO_2_ and dust emissions from secondary and tertiary industry decline markedly.

Coal is currently the main source of energy in Tongzhou District; it produces a great deal of SO_2_ and dust in the combustion process and is the major source of air pollution. To control air pollution, the Tongzhou district government adjusts the energy structure, relocates polluting enterprises, and installs high-efficiency desulphurization to the coal-fired boilers, which all improve the region's air quality significantly. The environmental air capacity of SO_2_ is 41.31 thousand t/a [38], which is greater than the SO_2_ emissions.

*(5) Solid Waste Emissions.* From the prediction results in Figure 10(g), the solid waste emissions of Tongzhou District in 2020 amount to 1.26 million tonnes, a slight decrease over 10 years; daily life solid waste continues to increase with the growth of the population and the improvement in living standards, increasing from 0.37 million tonnes in 2011 to 0.44 million tonnes in 2020. Owing to comprehensive utilization, the solid waste emissions from primary and secondary industry all decrease after 2011 to 0.73 and 0.04 million tonnes, respectively, in 2020. However, primary industry is still a major source for solid waste emissions, accounting for 58 percent of the region's emissions.

#### 4.2.2. Simulation Results of the Planning-Oriented Scenario

**Figure 11:**
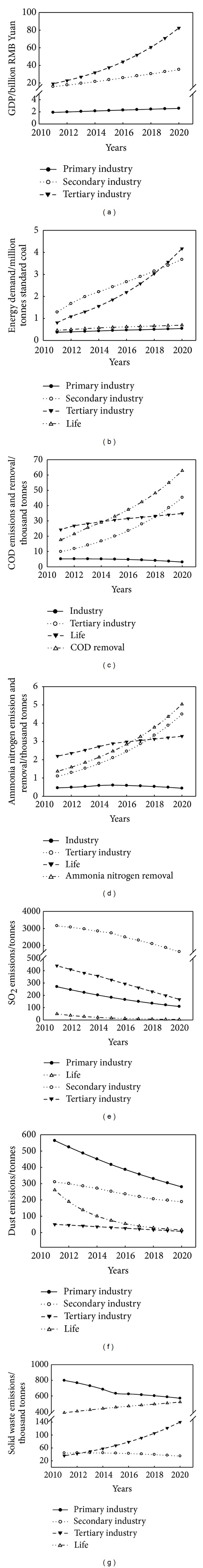
Simulation results under the planning-oriented scenario. (a) Economy development, (b) energy consumption, (c) COD emissions, (d) NH_3_-N emissions, (e) SO_2_ emissions, (f) dust emissions, and (g) solid waste emissions.

*(1) Economic Development.* As is shown in Figure 11(a), the GDP of Tongzhou District rises from 37.06 billion RMB Yuan in 2011 to 120.4 billion RMB Yuan in 2020 under the planning-oriented scenario. Efforts should be intensified to adjust the industrial structure in the next 10 years, raising the ratio of tertiary industry from 52 to 69 percent while reducing the ratios of primary and secondary industry from 5 to 2 percent and from 43 to 29 percent, respectively.

In the planning-oriented scenario, the government drives the expansion of the economy by increasing investment. The emphasis is on tertiary industry, urban industry, and modern manufacturing. However, the environmental costs are also shocking with the further development of the economy.

*(2) Energy Consumption.* From the prediction results in Figure 11(b), the total energy demand of Tongzhou District increases rapidly and will reach 9.08 million tonnes standard coal in 2020, 3.01 million tonnes more standard coal than the amount in the business-as-usual scenario. The energy consumption per unit of GDP in 2020 is 0.75 tce/10000 RMB Yuan. The energy consumption of tertiary industry increases the most quickly, to 4.17 million tonnes standard coal in 2020, accounting for nearly half of the region's total energy demand. Secondary industry also requires large quantities of energy, 3.68 million tonnes standard coal in 2020, 0.15 million tonnes more than the amount in the business-as-usual scenario. By 2020, primary industry and regional inhabitants consume, respectively, 0.08 and 0.17 million tonnes more standard coal in the planning-oriented scenario than in the business-as-usual scenario. The energy shortage would hinder regional social and economic development severely.

In the planning-oriented scenario, energy also faces a critical gap although the energy-intensive industries are restricted by industrial structure adjustment, such as the petrochemical and rubber and plastics industries. Thus, depending on technical advancements and either process or equipment modifications, it is suggested that energy consumption be decreased at the enterprise level.

*(3) Water Pollution Emissions.* The prediction results in Figures 11(c) and 11(d) indicate that COD and ammonia nitrogen production show relatively large increases over the next 10 years, respectively, 36.70 and 3.19 thousand tonnes more than the output in the business-as-usual scenario in 2020. However, the COD and ammonia nitrogen removal amounts also increase significantly compared with those under the business-as-usual scenario, by 39.35 and 3.31 thousand tonnes, respectively, in 2020. The final COD and ammonia nitrogen emissions are 20.54 and 3.18 thousand tonnes in 2020, 11 and 4 percent lower than those in the business-as-usual scenario. Tertiary industry has the highest emission proportions of COD and ammonia nitrogen, accounting for 54 and 55 percent, respectively, in 2020. However, the ratios of industrial COD and ammonia nitrogen water pollutants decline by, respectively, 4 and 5 percent in 2020.

In the planning-oriented scenario, with industrial structure adjustment and larger investments in sewage disposal facilities than before, the final water pollution emissions decline. However, the total existing pollution loadings also greatly exceed the environmental water capacity. Thus it is necessary to build more polluted water treatment facilities.

*(4) Air Pollution Emissions.* As shown in Figures 11(e) and 11(f), the SO_2_ and dust emissions in 2020 are 1.91 and 0.49 thousand tonnes, 0.01 and 0.20 thousand tonnes less than those in the business-as-usual scenario. The dust emission proportions of primary and secondary industry are very high, at 57 and 38 percent; however, the SO_2_ emission proportion of secondary industry is highest at 85 percent.

Because of the promotion of desulfurization and dedusting technology and the limits on high-pollution enterprises such as the petrochemical industry, SO_2_ and dust emissions begin to decrease.

*(5) Solid Waste Emissions.* As shown in Figure 11(g), the solid waste emissions of Tongzhou District increase slightly in the planning-oriented scenario. The total solid waste emissions reach 1.27 million tonnes in 2020, 0.01 million tonnes more than the amount in the business-as-usual scenario. Owing to improved agricultural solid waste utilization, solid waste emissions from primary industry reduce to 0.57 million tonnes in 2020, 0.16 million tonnes less than those in the business-as-usual scenario in 2020, alleviating the serious solid waste pollution, in particular, rural environmental pollution. However, primary industry is still a major source of solid waste emissions, accounting for 45 percent of the region's emissions in 2020. Solid waste emissions from secondary industry decrease slightly, to 2.91 thousand tonnes less than the amount in the business-as-usual scenario. However, with the rapid development of tertiary industry and the increase in people's living standards, solid waste emissions from tertiary industry and from daily life show large increases, respectively, 0.09 and 0.09 million tonnes greater than the amounts in the business-as-usual scenario.

Agricultural waste is the main source of biomass resource, but it is ineffective when used over a long period. Thus, primary industry has more potential to abate solid waste emissions.

#### 4.2.3. Simulation Results of the Sustainable Development Scenario

**Figure 12:**
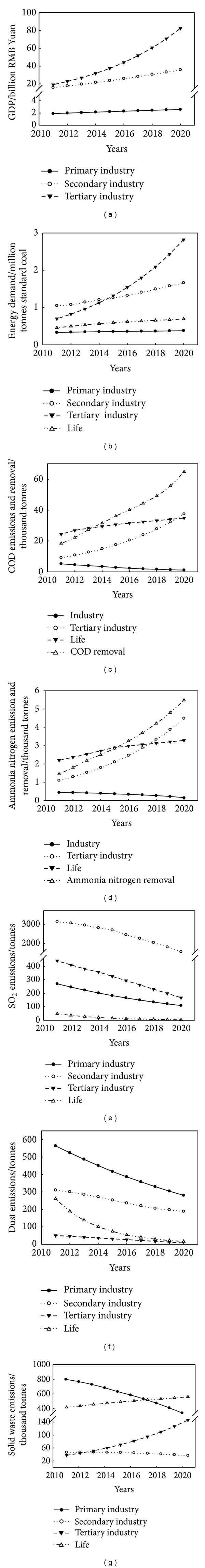
Simulation results under the sustainable development scenario. (a) Economy development, (b) energy consumption, (c) COD emissions, (d) NH_3_-N emissions, (e) SO_2_ emissions, (f) dust emissions, and (g) solid waste emissions.

*(1) Economic Development.* The population size and structure and the scale of the regional economy in the sustainable development scenario remain consistent with those in the planning-oriented scenario. As shown in Figure 12(a), the GDP of Tongzhou District reaches 120.41 billion RMB Yuan in 2020.

*(2) Energy Consumption.* The energy consumption prediction results are shown in Figure 12(b). The results show that the energy demand of Tongzhou District in the sustainable development scenario is significantly lower than that in the planning-oriented scenario. The total regional energy demand in 2020 is 5.56 million tonnes standard coal, a decrease of 39 percent compared with the demand in the planning-oriented scenario. The energy consumption per unit of GDP in 2020 is 0.46 tce/10000 RMB Yuan. Production energy consumption is reduced by improving industrial furnaces and making full use of industrial waste heat. Energy consumption by primary, secondary, and tertiary industry in 2020 is, respectively, 0.38, 1.67, and 2.82 million tonnes standard coal, which is 0.16, 2.01, and 1.35 million tonnes, respectively, less than the amounts in the planning-oriented scenario. The daily life energy demand is 0.69 million tonnes standard coal in 2020, the same as that in the planning-oriented scenario.

In the sustainable development scenario, energy consumption per unit of GDP also does not reach the requirements of the new town plan. To reduce energy consumption, it is first necessary to improve the energy efficiency standards for residential buildings and increase energy education. Second, it is possible to restrict the growth of high-energy-consumption companies further.

*(3) Water Pollution Emissions.* In the sustainable development scenario, the COD and ammonia nitrogen emissions from daily life and from tertiary industry account for considerable proportions of the total water pollution, but those from industrial sources have been gradually brought under control. In addition, the water pollution abatement capacity has been greatly enhanced by the expansion of sewage treatment plants and the improvement of centralized sewage treatment. From Figures 12(c) and 12(d), it can be found that the final ammonia nitrogen and COD emissions in the sustainable development scenario decrease by 22.94 and 39.18 percent in 2020 compared with those in the planning-oriented scenario. In 2020, the COD and ammonia nitrogen abatement rates reach, respectively, 82.95 and 69.07 percent of the emissions. However, COD and ammonia nitrogen production in daily life in 2020 is, respectively, 34.87 and 3.29 thousand tonnes, the same as the amounts in the planning-oriented scenario. Although the pollution discharge has been further controlled, COD and ammonia emissions are rising along with the population growth and the expansion of the production scale. The water pollution emissions are still over the limits.

At present, only part of the large living area and service establishments receive complete sewage treatment. Most untreated sewage is discharged directly into surface water, causing water pollution. It is therefore necessary to construct more urban sewage treatment plants and to expand the sewage pipe network. In addition, residential water reuse systems should be built to reduce wastewater discharge.

*(4) Air Pollution Emissions.* Through new technologies, new adjustments to the energy structure and new desires for cleaner air, dust and SO_2_ emissions decline markedly in the business-as-usual and planning-oriented scenarios. Thus, the air pollutant reduction potential is slight in the sustainable development scenario.

From Figures 12(e) and 12(f), it can be found that SO_2_ emissions in the sustainable development scenario decrease by 4 percent in 2020 compared with the emissions in the planning-oriented scenario. This is mainly owing to the relocation of polluting enterprises and the use of clean energy, such as electricity, natural gas, and solar.

*(5) Solid Waste Emissions.* From the prediction results in Figure 12(g), it can be seen that the total discharge of solid waste in Tongzhou District gradually decreases. The solid waste emissions in the sustainable development scenario decrease by 18 percent in 2020 compared with the amount in the planning-oriented scenario. Primary industry is the major source, but the emissions in 2020 are 0.23 million tonnes lower than they are in the planning-oriented scenario. The solid waste emissions of secondary and tertiary industry and daily life in 2020 are the same as the amounts in the planning-oriented scenario. As a result, and also owing to the growth of the population, the improved living standards, and the rise of the service industry, the amount of solid waste discharged from daily living and from tertiary industry is still large. It is therefore suggested that all urban garbage disposal should see further progress on the basis of present conditions.

## 5. Conclusion

In this study, the simulation results reveal the following. (1) Although it is impossible to achieve the economic objective of the Tongzhou new town plan under the business-as-usual scenario, there is rapid economic growth in Tongzhou District, which has a huge impact on energy and the environment. Following the current trends, energy consumption and pollution emissions would exceed the environmental carrying capacity. (2) In the planning-oriented scenario, the socioeconomic objectives of the Tongzhou new town plan can be achieved by increasing investment and adjusting the investment structure. However, the realization of the targets comes at the expense of energy consumption and environmental damage. This is mainly attributable to the rapid growth of the economy, which greatly increases energy demand and pollution emissions. (3) In reality, the energy consumption of Tongzhou District depends mainly on foreign supply. According to the Tongzhou new town plan requirements, energy consumption per unit of GDP in 2020 should reach 0.43 tce/10000 RMB Yuan. This requirement cannot be met in any of the three scenarios. The contradiction between energy supply and demand is constantly increasing. In another sense, the energy planning rules in the Tongzhou new town plan may be somewhat too rigid. (4) The results show that the water pollution problem in Tongzhou District is very serious. The COD and ammonia nitrogen emissions could not reach the requirements of the environmental water capacity even under the three scenarios. Thus, the key constraints to future socioeconomic development in Tongzhou District are energy shortages and water pollution. (5) In response, a series of measures should be passed to cut energy use and water pollution, including improving the energy efficiency standards for residential buildings, closing energy-wasting and highly polluting factories, and constructing more urban sewage treatment plants and residential water reuse systems.

## Figures and Tables

**Table 1:** Sensitive degrees resulting from the variations of input parameters.

Parameter	S (%)						
	2004	2005	2006	2007	2008	2009	2010
Industrial COD generation coefficient I01	0.60	0.65	0.65	0.70	0.70	0.75	0.80

Industrial solid waste generation coefficient I01	17.60	15.05	12.85	11.00	9.45	8.15	7.05

Industrial COD generation coefficient I05	243.10	243.75	245.95	249.75	255.45	261.10	268.60

Industrial ammonia nitrogen generation coefficient I05	18.75	18.65	18.75	19.00	19.40	19.65	20.05

Industrial energy consumption per unit of GDP I05	5.80	5.45	5.15	4.85	4.60	4.40	4.20

Industrial ammonia nitrogen generation coefficient I07	1.15	1.20	1.25	1.35	1.45	1.55	1.65

Industrial ammonia nitrogen generation coefficient I08	262.40	280.95	299.90	319.30	339.30	353.40	366.25

Industrial solid waste generation coefficient I08	65.25	67.50	69.25	70.60	71.55	72.15	72.35

Industrial COD generation coefficient I09	1.95	2.05	2.20	2.35	2.55	2.70	2.95

Sulfur conversion rate of industrial coal I10	4.70	4.60	4.50	4.45	4.40	4.40	4.40

Industrial energy consumption per unit of GDP I10	11.30	10.95	10.60	10.30	10.05	9.85	9.65

Industrial energy consumption per unit of GDP I11	12.25	12.15	12.05	12.00	12.00	11.95	11.95

Sulfur conversion rate of industrial coal I14	0.80	0.75	0.70	0.65	0.65	0.60	0.55

Sulfur conversion rate of industrial coal I15	2.70	2.70	2.65	2.65	2.65	2.60	2.60

Industrial solid waste generation coefficient I15	1.90	1.95	2.05	2.10	2.20	2.30	2.40
